# Analysis of the suspected cancer‐causing potassium bromate additive in bread samples available on the market in and around Dhaka City in Bangladesh

**DOI:** 10.1002/fsn3.2338

**Published:** 2021-05-24

**Authors:** Syed Sadman Mahmud, Mukta Moni, Abu Bin Imran, Tahmina Foyez

**Affiliations:** ^1^ Department of Pharmaceutical Sciences School of Health and Life Sciences North South University Dhaka Bangladesh; ^2^ Department of Chemistry Faculty of Engineering Bangladesh University of Engineering and Technology Dhaka Bangladesh

**Keywords:** bread, carcinogenic, potassium bromate, promethazine, spectrophotometry

## Abstract

Bread is one of the most popular foods consumed worldwide. It is a very popular foodstuff consumed in almost every house in Bangladesh as breakfast. Bread is prepared predominantly from flour to meet the daily carbohydrate demand and enhances its overall nutrition value using various ingredients. Potassium bromate (KBrO_3_) is an alluring additive to improve bread quality by bread makers. But due to the well‐known toxic and carcinogenic effect, certain levels of KBrO_3_ residue are not suitable for bread, and it is therefore forbidden in many countries. The key objective of this study is to evaluate the safety status of bread in Dhaka City and its proximity to Bangladesh. Twenty‐one randomly collected bread samples were tested in this study from different bakeries or shops in and around Dhaka City. The levels of KBrO_3_ were analyzed spectrophotometrically, and the maximum concentration found in the bread sample was 9.29 μg/g. A total of 67% of collected bread samples showed elevated levels of KBrO_3_ relative to the allowable amount prescribed by various Food and Drug Administration worldwide. KBrO_3_ is toxic to consumers and could endanger their health over continuous regular consumption and thus need to be monitored strictly.

## Graphical abstract

A total of twenty one randomly collected bread samples were tested in this study from different bakeries or shops in and around Dhaka city. The levels of KBrO3 were analyzed spectrophotometrically and the maximum concentration found in the bread sample 9.29 μg/g. A total of 67% of samples showed elevated levels of potassium bromate, which is extremely toxic to consumers and could endanger their health over continuous regular consumption thus need to be strictly monitored.

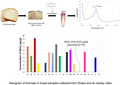

## 1 INTRODUCTION

**FIGURE 1:**
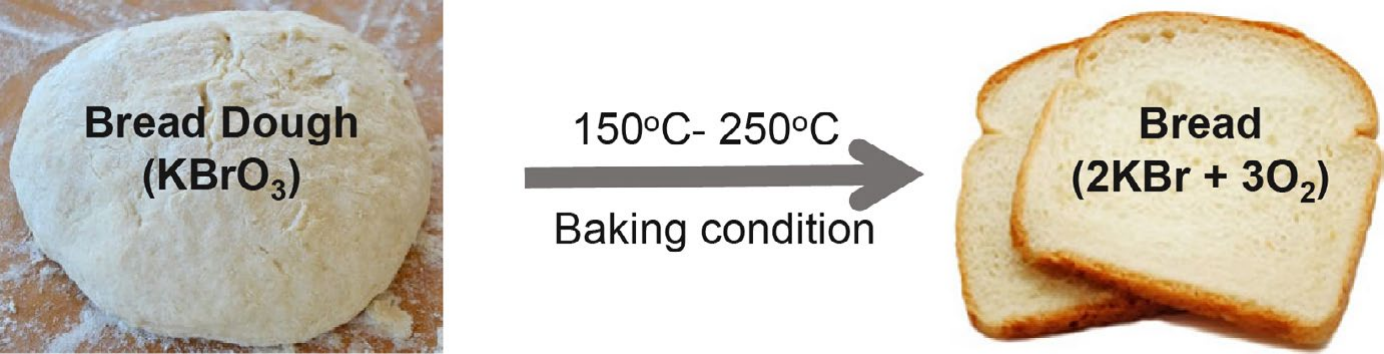
KBrO_3_ of bread dough completely reduces to KBr when 150°C‐250°C is maintained during baking

A fundamental human right is to access safe food. Bread is an essential food that does not need additional preparation before consumption (Afolabi et al., 2015). In Bangladesh, bread is primarily consumed by all classes of people during breakfast, evening snacks, and school tiffin. It is affordable and one of the most familiar convenient foods available for consumption. It is consumed as a portion of favorite food made from wheat with low protein. Flour, salt, yeast, water, and flour improvers are the major constituents of bread (Ojeka et al., (2006)). Bread is categorized as a dietary source of carbohydrates, vitamins, and some minerals such as selenium, copper, magnesium, and dietary fiber (Dietary guidelines for Americans, 2010). Several dough conditioners mature the flour, stabilize the bread dough's gluten network, and increase its elastic properties. KBrO_3_ is a popular food enhancer that has been used by the baking industry for over a century. It occurs as a white crystalline solid with almost no flavor and odor and is freely soluble in water. KBrO_3_ makes the bread stronger, increases its volume, and enhances its texture (Gandikota, 2005). It oxidizes the sulfhydryl (R‐SH) groups into disulfide bonds of the gluten protein of flour, which keeps the dough more elastic by increasing the volume and softness of bread to maintain the carbon dioxide gas created by the yeast. In baked products, toxic KBrO_3_ gets reduced to potassium bromide (KBr) (Figure 1), which is thought to be harmless in the finished baked end product ( Cogswell, 1997; Emeje et al., 2010).

However, if an excessive amount of KBrO_3_ is used, or if the bread is baked for a shorter period of time, or if the process is not carried out at a sufficiently high temperature, the remaining quantity of KBrO_3_ can be noticed and provide the toxic effect (Bushuk & Hlynka, 1960). The use of bromate has been the topic of ongoing controversy. Many scientists reported in the early 1990s that the use of KBrO_3_ is likely to be safe as a bread additive, ensuring it is completely degraded during baking to less dangerous products. But when the World Health Organization (WHO) declared in 1993 that large quantities of residual bromate were observable in 75% of the loaves tested in the UK, this was followed by its prohibition in the UK (Joint & Additives WECoF, Organization WH,,). As KBrO_3_ is detrimental to health and should be prohibited to use in bread. It is reported that continuous consumption of KBrO_3_ can result in sore throat, diarrhea, nausea, vomiting, abdominal pain, low blood pressure, depression, thrombocytopenia, cancer, and other health problems (Ajarem et al., 2016; Atkins, 1993; Robert & William, 1996). In living organisms as well as in humans, nephrotoxic and toxic effects of KBrO_3_ have been observed. It is a genotoxic carcinogen capable of causing renal, mesothelioma, and thyroid follicular cell tumors in rats (Kurokawa et al., (1990)). Due to health‐related issues, the use of KBrO_3_ is now prohibited in many countries. Its consumption in the United States was reduced to 75 μg/g of flour (U.S Food and drug administration., (2018)). In 1999, the International Agency for Research on Cancer (IARC), associated with the WHO, classified KBrO_3_ as a possible carcinogen for humans. While it is still used in the United States where the maximum permissible level set by the U. S. Food and Drug Administration (FDA) is 50 μg/g of flour and 10 μg/g of flour in Japan ( Abu‐Obaid et al., 2016). During the heating process, bromate should be almost completely reduced to bromide, the harmless form of the oxidizer. The bakery industry needs to follow policies and procedures to minimize any possible residues of bromate in the baked goods to a safe level established by the FDA risk analysis at 0.02 μg/g (American Bakers Association (ABA),; Ekop et al., 2008; ). In addition to its carcinogenicity, KBrO_3_ has been found to impact the nutritional consistency of bread. The major vitamins in bread, including vitamin A2, B1, B2, and niacin, are depleted using excess KBrO_3_ (Okafor et al., 2011).

In Bangladesh, as per the Bangladesh standards and testing institution (BSTI) regulations in 2018, the use of KBrO_3_ is not permissible (BSTI). Despite the option of many other non‐toxic substitutes, many bakers still use KBrO_3_ to prepare their bread to jeopardize public life. The current spectrophotometric analysis aimed to examine the residues of KBrO_3_ in finished bread in Dhaka and its nearby cities, recognizing the possible threat posed by these agents so that the related authorities can take necessary and drastic measures to limit the use of this food poison.

## 2 EXPERIMENTAL

### 2.1 Materials and sampling

All reagents used were of analytical grade obtained from recognized chemical companies. For example, hydrochloric acid (HCl) from Thermo Fisher Scientific, USA, potassium bromate from Sigma‐Aldrich, Germany, and promethazine hydrochloride from Sigma‐Aldrich, Germany, were purchased and used as received. The deionized water was used throughout the experiment unless otherwise noted. A total of randomly chosen 21 bread samples of different brands were purchased from various bakeries, retailers located in and around Dhaka City, Bangladesh. All the bread samples were manufactured from wheat flour and denoted from the letters A to U before analysis.

### 2.2 Sample preparation

First, the hard surrounding portions were cut off from the bread with a clean and sharp knife. The central white portion of each bread was dried in the oven for 1 hr at 85°C and was finely ground manually using mortar and pestle. The powdered and dried bread was stored in sealed containers for analysis (Alli et al., 2013).

### 2.3 Spectrophotometric analysis

**FIGURE 2:**
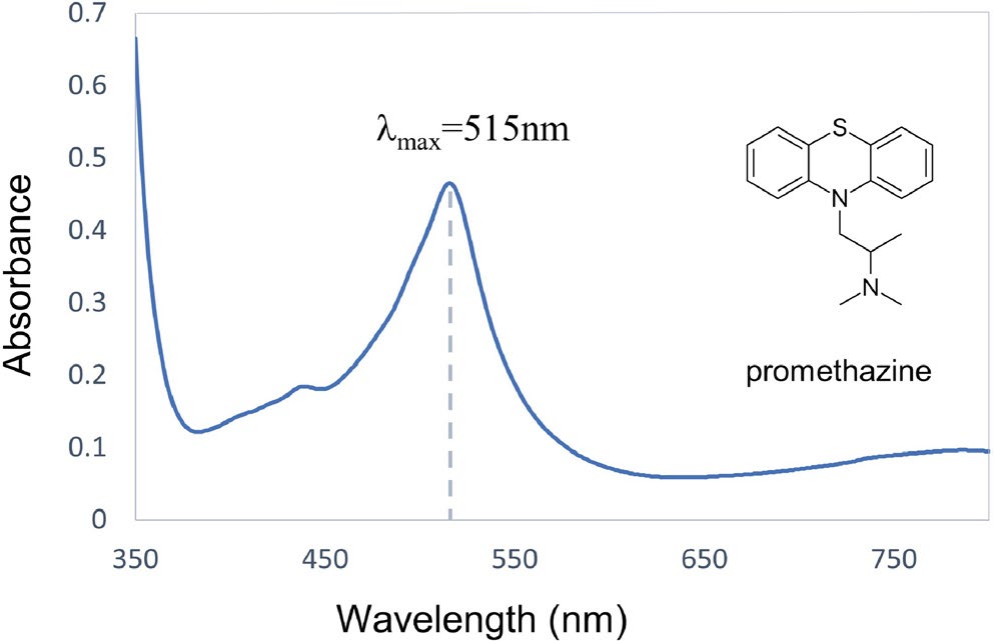
UV‐vis spectrum of oxidizing product between promethazine hydrochloride and KBrO_3_ (3µ g/mL) solution in deionized water. The wavelength corresponding to absorption maximum (λ_max_) is observed at 515 nm

**FIGURE 3:**
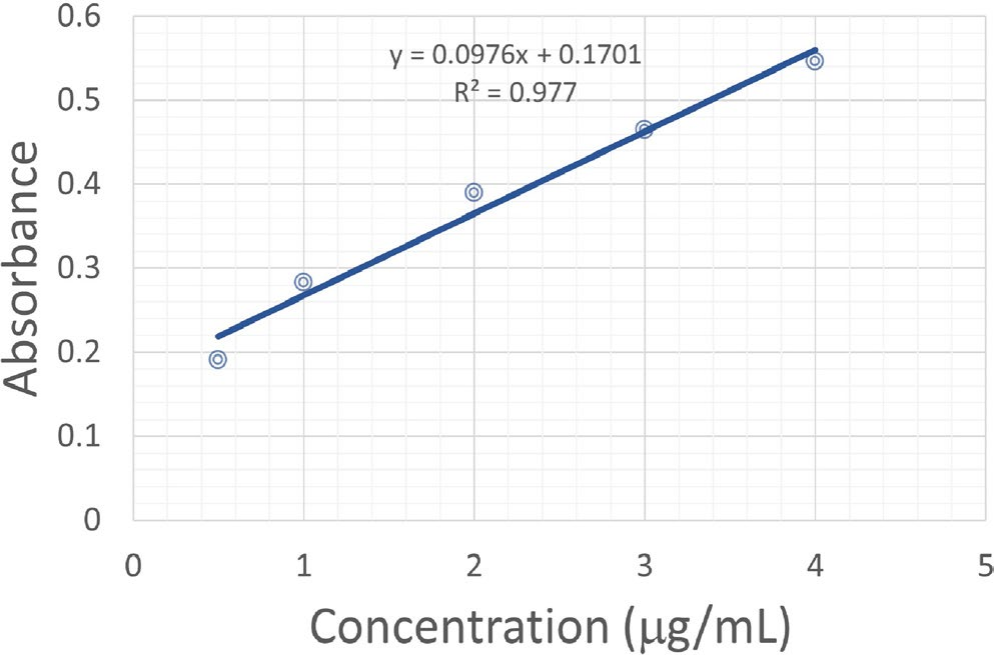
Standard calibration curve of KBrO_3_ for spectrophotometric determination of bread sample

For the quantitative evaluation of KBrO_3_ in the bread samples, the spectrophotometric method reported by El Harti et al. was used (El‐Harti et al., 2011). Briefly, a stock solution of KBrO_3_ (1,000 μg/mL) was prepared in deionized water from which different intermediate stock standard solutions were prepared. Different aliquots of KBrO_3_ solution were put into 10‐mL volumetric flasks and added 2.0 ml of 0.01 M promethazine and 0.2 ml of 12 M HCl. The deionized water was then added up to the mark of the volumetric flask to prepare the desired solution. The solution was shaken well for one minute, and the absorbance was measured against the wavelength corresponding to absorption maximum (λ_max_) at 515nm (Figure 2). A standard curve was plotted, and the regression equation of calibration plot was calculated by the least squares method for further analysis of the bread samples (Figure 3).

For bread sample analysis, 1 g of each bread powdered was taken in a clean centrifuge tube and 20 ml of deionized water was added. The mixture was vortexed for about 2 min and then filtered. The filtrate bread solution was then added to the 10‐mL volumetric flask and was applied with 2.0 ml of 0.01 M promethazine and 0.2 ml of 12 M HCl. The mixture was then shaken for 1 min, and the absorption of the pink‐colored solution was measured spectrophotometrically. The concentration was estimated from the linear regression curve derived from standard KBrO_3_ solutions.

## 3 RESULTS AND DISCUSSION

**FIGURE 4:**
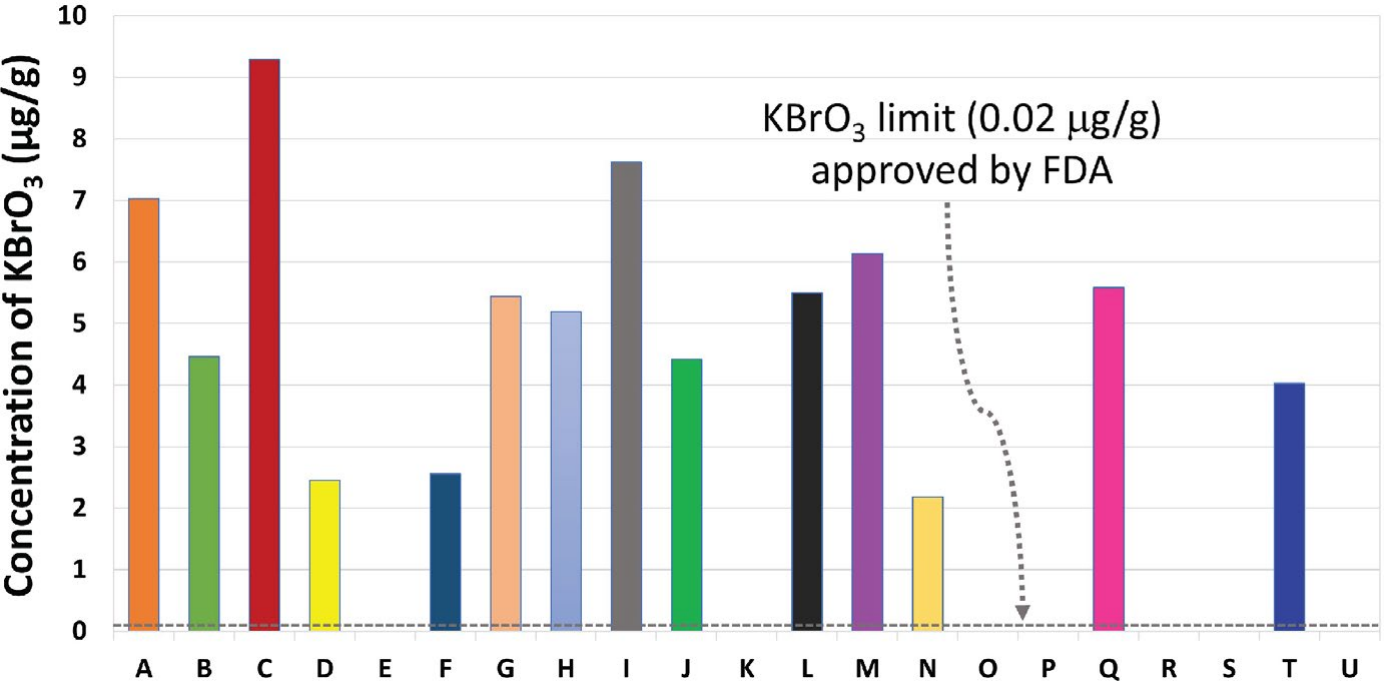
Histogram of bromate in bread samples collected from Dhaka and its nearby cities

The presence of KBrO_3_ was spectrophotometrically analyzed on bread samples of various brands. Promethazine is a 10‐[2‐(dimethylamino)propyl]phenothiazine monohydrochloride of phenothiazine compound. It is easily oxidized in acidic medium with a number of oxidants, for example, KIO_3_, KBrO_3_, KIO_4_, NaNO_2_, H_2_O_2_, chloramine T, K_2_Cr_2_O_7_, NH_4_VO_3_, FeCl_3_, and HNO_3_ with the formation of colored compounds (Girard et al., 1987; Hanson & Norman, 1973; Kojlo et al., 2001). The sulfur atom in the phenothiazine is very susceptible to oxidation, and the oxidation products are colored free radicals with absorption maxima at 500–640 nm. The free radicals are stable in acidic media, and the sulfur bridge makes resonance to stabilize the product. Phenothiazines are reversibly oxidized to a colored free radical or semiquinone with KBrO_3_ and further oxidized irreversibly to a colorless sulfoxide. The heterocyclic centered radical cations in general produce red‐pink color, subsequent oxidation such as 4‐hydroxy‐ 3‐ oxo‐ 3H‐ phenothiazine −5 ‐oxide, thus promethazine oxides with bromate to produce a red‐pink with absorption maxima at 515 nm. Spectrophotometrical quantitative analysis for most of the samples exhibits positive results (Table 1 and Figure 4). The highest concentration of 9.29 μg/g was found in sample C which was collected from Mohammadpur, Dhaka. The KBrO_3_ residue was not detected in the samples E, K, O, P, R, S, and U. The other anions including Cl^‐^, Br^‐^, I^‐^, NO_3_^‐^, and NO_2_^‐^ present in breads had no interference with bromate in the spectroscopic measurements.

The result obtained from the bread analysis showed that a great number of bread makers use KBrO_3_ as a bread improver. Bakeries and confectioneries are using KBrO_3_ in their bread manufacturing for economic benefit. It is used as a dough enhancer, and it has a pronounced action in maintaining the size, color, and texture of a bread. The more the quantity, the more appealing is the bread to the consumer.

**TABLE 1:** Quantitative estimation of the amount of KBrO_3_ observed in various bread samples collected mostly from different regions of Dhaka and its nearby cities

Sample code	Retail outlet	Location of retail outlet	Sample collection date	Concentration of KBrO_3_ in bread samples (μg/g)
A	Open market	Mymensingh	06‐Apr−19	7.03
B	Open shop	Narayanganj	07‐Apr−19	4.46
C	Open shop	Mohammadpur, Dhaka	11‐Apr−19	9.29
D	Open shop	Rampura, Dhaka	07‐Apr−19	2.45
E	Open market	Moghbazar, Dhaka	04‐Apr−19	Not detected
F	Super market	Keraniganj, Dhaka	08‐Apr−19	2.56
G	Open shop	Rampura, Dhaka	07‐Apr−19	5.44
H	Open shop	Narayanganj	07‐Apr−19	5.19
I	Open shop	Mirpur, Dhaka	11‐Apr−19	7.62
J	Super market	Jhenaidah	07‐Apr−19	4.42
K	Open market	Jhenaidah	11‐Apr−19	Not detected
L	Super market	Jhenaidah	08‐Apr−19	5.5
M	Open shop	Siddheswari,, Dhaka	06‐Apr−19	6.14
*N*	Super market	Rampura, Dhaka	07‐Apr−19	2.19
O	Super market	Kalabagan, Dhaka	06‐Apr−19	Not detected
P	Super market	Mirpur, Dhaka	06‐Apr−19	Not detected
Q	Open shop	Narayanganj	07‐Apr−19	5.58
R	Open market	Dhanmondi, Dhaka	12‐Apr−19	Not detected
S	Open market	Purana Paltan, Dhaka	03‐Apr−19	Not detected
T	Super market	Mohammadpur, Dhaka	12‐Apr−19	4.03
U	Super market	Kalabagan, Dhaka	06‐Apr−19	Not detected

There has been a serious argument over the use of KBrO_3_. Many scientists indicated that KBrO_3_ was likely to be harmless as a bread additive because the compound was broken down to negligible amounts during baking (Akunyili, 2004). However, in 1993, the Food and Drug Administration in America investigated some breads and found that all of the breads contained significant bromate concentrations (C.S.P.I., 2004). The breads tested by the UK Science Laboratory in 1989 had considerable residual bromate ([W.H.O., 1994). This was followed by its ban in Britain and many other countries. According to the Centre for Science and Environment (CSE) study report in 2016, the use of KBrO_3_ as a flour treatment agent had been forbidden in many countries around the world, such as the United Kingdom (1990), Nigeria (1993), Canada (1994), Sri Lanka (2001), Brazil (2001), Columbia (2002), China (2005), and India (2016). Despite the ban, Emeje et. al. reported that 92 percent of Nigerian bread samples contained KBrO_3_ (Emeje et al., 2010). The state of California needs a warning label to contain food containing KBrO_3_. The varying residual amounts of KBrO_3_ in the finished products may arise due to bread baking for a shorter time period, or the process is not being conducted at a high enough temperature. The persistence of KBrO_3_ may also occur in the bread due to the excess use of the KBrO_3_ (Bushuk & Hlynka, 1960). In Bangladesh so far, there is no report regarding the scenario of the presence of bromate in bread.

After absorption into the bloodstream, KBrO_3_ is converted into oxides and radicals. These highly reactive ingredients may affect DNA and play a role in developing cancer. Scientists noted damages like this in human liver and intestine cells that resulted in DNA strands splitting and chromosome damage caused by exposures to KBrO_3_ (Geter et al., 2006; Zhang et al., 2011). It can also damage the genetic material in cells. Researchers also reported substantial damage to cell membranes of lysosome, which are the small intracellular bodies responsible for essential cell functions such as cellular digestion. Models of the interaction between DNA damage and KBrO_3_ indicate a consistent low‐dose linear response, which means that the amount of DNA damage observed is proportional to the amount of KBrO_3_ ingested (Spassova et al., 2013).

In consideration of the data showing that KBrO_3_ can be genotoxic and carcinogenic, foods containing the KBrO_3_ should be avoided. Although the lethal dose of KBrO_3_ in humans has not been precisely determined, it is estimated to be approximately 5 to 500 mg/kg of body weight (Kurokawa et al., 1990). The bakeries should be looking to manufacture their baked products using better natural methods and materials (Cranton 2004). Ascorbic acid or vitamin C is considered a safe alternative to KBrO_3_. Glucose oxidase is an additional choice approved by the Food Safety and Standards Authority of India (FSSAI) in 2015.

## 4 CONCLUSIONS

In the current study, KBrO_3_ was found to be approximately above its acceptable level in 67% of collected bread samples in and around Dhaka City. Most of the bread samples had a high KBrO_3_ content, which is very harmful to health. The price or status of the bread samples did not influence the amount of KBrO_3_ present. There is indeed a need for more aggressive supervision by regulatory authorities in order to ensure the safety of customers. The government agencies are assigned to educate people on the use of hazardous KBrO_3_ as a food additive. The use of natural dough enhancers should be promoted, such as vitamin C powder, egg, apple, sauce, and so on.

## CONFLICT OF INTEREST

The authors declare no conflict of interest.

## DATA AVAILABILITY STATEMENT

Data available on request from the authors

## ETHICAL APPROVAL

This article does not contain any studies with human participants or animals performed by any of the authors.
